# From Jenner to genomics: the unfolding future of vaccinology

**DOI:** 10.3389/fimmu.2026.1860464

**Published:** 2026-06-09

**Authors:** Rabab Batool

**Affiliations:** Centre for International Health, University of Otago, Dunedin, New Zealand

**Keywords:** mRNA vaccines, DNA vaccines, cancer immunotherapy, viral vectors, mucosal immunity, vaccine delivery systems

## Abstract

The development of the smallpox vaccine two centuries ago laid the foundation for modern vaccinology. Smallpox, once a major killer in human history, was eradicated globally in 1980. This breakthrough encouraged the development of contemporary vaccine technologies, including inactivated, recombinant, viral vector, and mRNA platforms. Owing to developments in genetics, bioinformatics, and molecular biology, personalised vaccines have been made possible. Advances in the field of immunology facilitated an accelerated response to the COVID-19 pandemic. This perspective synthesises key developments and technological advances in the field of global immunisation. The author sheds light on emerging tools, transformative platforms, and long-term directions in vaccine science research.

## Historical foundations of vaccinology

Over two centuries ago, the first vaccine against smallpox was introduced. Smallpox is considered a major killer in human history. The deleterious disease had a case-fatality rate of approximately 50% among those infected. Before the development of the vaccine, smallpox prevention relied on variolation, a hazardous procedure that originated in Asia and Africa. Variolation was performed by deliberately exposing healthy individuals with powdered smallpox scabs or fluid from pustules of infected individuals, through superficial scratches in the skin. In 1796, the first smallpox vaccine, made from cowpox virus, provided a safer alternative and laid the groundwork for immunisation efforts (1). The widespread coverage of the vaccine led to the global eradication of smallpox in 1980. To date, smallpox is the only human infectious disease to have been eradicated globally (2).

The eradication of smallpox is a significant milestone in the history of immunisation. This breakthrough motivated numerous scientists to develop different vaccine platforms. The first inactivated (killed) whole-cell vaccine was developed for typhoid and cholera bacteria in 1896, followed by a viral vector vaccine for Ebola, and the mRNA vaccine for COVID-19 in 2019 and 2020, respectively. (3). Recombinant vaccines utilise genetic material from pathogens to produce specific antigens. Instead of using a whole, live, or killed virus, the specific gene that codes for a harmless surface antigen is isolated and inserted into a host cell (such as yeast or bacteria) to manufacture the antigen in large quantities safely. Compound vaccines incorporate multiple antigens to provide broader protection. Viral vector vaccines employ harmless viruses to deliver genetic material, such as Ebola and COVID-19 vaccines. These diverse methodologies are broadening our vaccine arsenal and influencing the future of infectious disease prevention and treatment. Advancements in new technologies and our improved understanding of immunological responses have ushered in a new era of vaccinology (4).

## Evolution of vaccine platforms

The future of vaccine science appears exceptionally promising. Today, we possess tools that were unavailable 15 or 20 years ago. We can comprehend, analyse, and unlock the complexities of the human immune system. Our knowledge has expanded, and we benefit from enhanced technologies in DNA sequencing and multi-omics. With high-performance computing and cloud computing, massive biological datasets (like DNA or protein sequences) can be analysed in the blink of an eye. We stand on the cusp of a golden age in vaccinology. We have reasonably addressed some of the less complex challenges such as measles and rubella. Currently, we confront some of the most complex challenges that have long eluded resolution. However, with modern technologies and recent advances, it is anticipated that these will soon be overcome.

One of the main challenges in vaccines for infectious diseases is designing them to be durable, provide long-lasting protection, and cover multiple variants. Today, our understanding of the immune system is sufficiently advanced. We now comprehend immune memory and its activation through vaccinology, thereby enabling long-lasting protection. Some of the recent examples are influenza, COVID and respiratory syncytial virus (RSV) vaccines. RSV is one of the most common reasons for hospital admission of children under five years of age (5). Current insights into RSV pathogenesis and immunity provide the basis for protecting older adults, high-risk younger individuals, pregnant women as well as newborns (6).

The immune system is inherently the body’s most potent mechanism for eliminating cancer. However, harnessing the full potential of the immune system for targeted cancer eradication with minimal side effects has remained a major challenge. Traditional treatments, such as chemotherapy, are often associated with significant toxicity and limited efficacy. The field has long struggled to translate immunological insights into effective therapies. Recent advances drawing on progress in vaccine development and infectious disease research, fortunately, now place us in a position to more effectively engage the body’s defence against cancer. The fusion of knowledge and technological innovation heralds a transformative era in immuno-oncology (7).

We are approaching a significant milestone in our ability to effectively combat infectious diseases. Many vaccines for infectious diseases do not necessarily prevent infection outright. Rather, the primary function of vaccines, which is of critical importance, is to reduce morbidity and mortality (8). The subsequent objective is to prevent transmission. Currently, some vaccines do not achieve sterilising immunity. The vaccinated individuals can still become infected, albeit to a lesser extent. Vaccines may help clear the infection earlier, reduce the severity of illness and the duration of infectiousness. If the duration and intensity of infectiousness are diminished, the probability of transmitting the disease to vulnerable populations such as infants, household members, or the elderly would decrease substantially (9, 10). Moreover, the development of a universal influenza vaccine, which may not be perfect but effectively prevents hospitalization and death, should be a primary goal. Ultimately, the prevention of transmission remains a crucial objective in our efforts to control infectious diseases (11).

## Cancer immunotherapy

The major challenge in the development of cancer vaccines is the identification of tumour-specific antigens. Neoantigens are highly specific to cancer cells, making them an ideal target for vaccines. They allow the immune system to recognise cancer as foreign and eventually elicit an effective immune response. Unlike pathogens, which the immune system readily recognises as foreign, cancer arises from the body’s own tissues. It is therefore inherently challenging for the immune system to detect cancer cells. Effective cancer vaccines must therefore target antigens uniquely expressed in tumour cells but absent from normal tissues (12). These antigens typically result from DNA mutations accumulated during rapid cancer cell proliferation, creating neoantigens that can be recognised as foreign. Notably, these mutations are often unique to each individual’s cancer. Therefore, cancer vaccines must be personalised. Advances in genomic sequencing, RNA/DNA platforms, and rapid vaccine production have made it feasible. Now, bespoke cancer vaccines can be generated in real time, transforming personalised immunotherapy from concept to reality (13, 14).

Effective antigen presentation is crucial for inducing robust local and systemic immunity. Administration techniques are a key area of interest, particularly in the context of respiratory viruses. Respiratory infections do not occur in the arm but rather in the nasal passages and lungs. Therefore, generating and sustaining local immune responses at mucosal surfaces or preferably the site of infection is critical. However, conventional intramuscular injections are limited in this regard (15). Following intramuscular administration, vaccine antigens drain to regional lymph nodes near the injection site, which does not efficiently prime immune responses in the lungs (16). This limitation arises from the anatomical organisation of the immune system. The lymph nodes are localised to different parts of the body, and antigens delivered via an intramuscular injection primarily drain to the lymph nodes near the injection site, rather than to the lungs or respiratory mucosa (17).

## Mucosal immunity and challenges in vaccine delivery

Innovations such as RNA-based vaccines, self-amplifying RNA vaccines, and certain adenovirus-vector vaccines delivered intranasally allow immune responses to be established directly at the relevant mucosal surfaces (18). A major focus among vaccinologists is delivering vaccines through the site of natural pathogen entry. For instance, for enteric or intestinal pathogens, oral vaccines are used, as exemplified by typhoid and rotavirus vaccines to prevent infant diarrhoea; for respiratory pathogens, intranasal delivery targets the nasal mucosa, as in influenza and COVID-19 vaccines (19). Similar approaches are being explored for pertussis to present antigens in a manner that closely mimics natural infection, thereby establishing a mucosal imprint that can be leveraged in subsequent immune responses (20).

In the future, vaccination strategies may induce local mucosal immunity in low-risk individuals. This can generate strong local antibody responses even if systemic antibody levels are modest. Alternatively, individuals entering higher-risk periods may then receive injectable vaccines to boost systemic immunity, achieving both local and systemic protection (21).

The primary challenge lies in presenting antigens to immune cells in a manner that elicits effective responses. While intramuscular delivery is well characterised, achieving localised immune responses that optimise both efficacy and safety remains challenging. Advances in platform design, including vector and adjuvant technologies, are therefore critical and carry broad implications for both pathogen and cancer vaccines (22). An earlier example is the rotavirus vaccine developed in the late 1990s for paediatric use. The vaccine, in rare cases, caused intussusception due to local inflammation, leading to its withdrawal. Insights from such experiences have informed the rational design of safer and more efficacious vaccines today (23).

## The future of vaccinology: integrated and personalised approaches

Sustained investment in cutting-edge technologies and vaccine research is pivotal to unlocking the full potential of modern vaccinology. The first recorded case of COVID-19 was in 2019. By December 2020, vaccines had already been developed and made available. This may appear unprecedented to those unfamiliar with the decades of foundational research that made such rapid vaccine development possible. The development of these vaccines relied heavily on funding through the U.S. federal government, particularly the National Institutes of Health (NIH), which supported the development of RNA technologies. Early sequencing of viral isolates was critical for identifying the precise antigens required for vaccine design. All of this was built upon more than 50 years of basic science and fundamental research, which provided the foundation to produce a COVID-19 vaccine in record time.

Messenger RNA (mRNA) technology has been under investigation since the 1960s. Initial trials explored its potential in preventing diseases such as Ebola. With the emergence of COVID-19, these early efforts were redirected to address the pandemic. Additionally, mRNA vaccines have been tested in clinical trials for other infectious diseases, including influenza, respiratory syncytial virus (RSV), and Zika virus (24).

mRNA technology has been investigated since the 1970s for developing vaccines against cancers such as melanoma and lung cancer. It has also been explored for other cancer treatments. The technology has enabled breakthroughs in preventing the recurrence of aggressive cancers after surgery and in training the body to attack some cancers before they can grow. Messenger RNA (mRNA) vaccines have transformed modern vaccinology and have revolutionised the COVID-19 response, with potential beyond the pandemic (24).

BioNTech, the company that developed RNA vaccines for SARS-CoV-2, originally focused on cancer vaccines. The goal was to create individualised RNA vaccines for cancer by leveraging the platform’s rapid production capabilities (25). Before the pandemic, a clinical trial using RNA vaccines was underway to treat pancreatic cancer patients. Consequently, some of the earliest recipients of RNA vaccines in the U.S., in December 2019, were cancer patients rather than SARS-CoV-2 patients (26). At the start of the pandemic, BioNTech shifted its platform to infectious disease vaccines. RNA-based vaccines offer the advantage of rapid development and can be tailored to specific diseases and new variants. This feature makes them critical for both personalised cancer therapy and pandemic response. Furthermore, mRNA technology is also being explored for cancer immunotherapies and other treatments (27).

Building on advances in RNA and other vaccine platforms, DNA vaccines, also known as plasmid vaccines, represent another innovative approach. The DNA vaccines function by delivering short DNA sequences into host cells that encode instructions for producing antigens derived from a specific virus or bacterium (28). Following administration, host cells transcribe and translate the introduced DNA, leading to the production of these antigens. This process elicits an immune response, enabling the immune system to recognise and mount protection against the pathogen upon subsequent exposure (29).

One of the potential benefits of this approach is that the immune system’s response can be much stronger than with other types of vaccine. DNA vaccines are also more stable and easier to produce than mRNA vaccines. Unlike mRNA vaccines, DNA vaccines are feasible for global distribution and accessibility due to their thermostability and storage convenience, as they do not require strictly subfreezing temperatures (29, 30).

**Table 1** summarises the evolution of vaccine technologies from classical whole-organism vaccines to modern recombinant, vector-based, and nucleic acid platforms, underlying principles of each type of vaccine, representative licensed vaccines, and landmark milestones.

**Table 1:** Evolution of vaccines from classical to modern platforms.

Vaccine type	Platform principle	Licensed vaccines using this technology	First introduced (landmark vaccine)
Live attenuated (weakened/inactivated)	Pathogens are weakened/inactivated so they replicate poorly while retaining immunogenicity and inducing durable immune responses.	Measles, mumps, rubella, yellow fever, influenza (live/inactivated), oral polio (OPV), typhoid, Japanese encephalitis, rotavirus, BCG, varicella-zoster	1798 (smallpox)
Killed whole-organism	Whole pathogens are grown in culture and killed using formalin/heat presenting full antigenic repertoire without replication.	Whole-cell pertussis, inactivated polio (IPV), influenza, Japanese encephalitis, hepatitis A, rabies	1896 (typhoid)
Toxoid vaccines	Bacterial toxins are chemically inactivated/detoxified while preserving immunogenic epitopes to induce anti-toxin immunity.	Diphtheria, tetanus	1923 (diphtheria)
Subunit vaccines (purified protein, recombinant protein, polysaccharide, peptide)	Specific antigenic components are purified from pathogens or produced via recombinant DNA technology; may include polysaccharides or peptides.	Hepatitis B, pertussis (acellular), influenza (subunit), meningococcal, pneumococcal, typhoid (Vi polysaccharide), hepatitis A	1970 (anthrax)
Virus-like particle	Self-assembling viral structural proteins form empty virus-like shells without genetic material, mimicking native virus structure.	Human papillomavirus (HPV)	1986 (Hepatitis B)
Outer membrane vesicle (OMV)	Naturally shed bacterial membrane vesicles containing outer membrane proteins and lipooligosaccharides used as immunogens.	Group B meningococcal (MenB)	1987 (MenB)
Protein–polysaccharide conjugate	Poorly immunogenic polysaccharides are chemically linked (conjugated) to a carrier protein to induce T-cell–dependent immune response.	Haemophilus influenzae type b (Hib), pneumococcal, meningococcal, typhoid conjugate vaccines	1987 (Haemophilus influenzae type b conjugate vaccine)
Viral vectored	A harmless virus (e.g., adenovirus, VSV) is engineered to deliver genetic code encoding antigen into host cells.	Ebola virus disease (e.g., rVSV-ZEBOV), COVID-19 (adenoviral vectors)	2019 (Ebola)
Nucleic acid vaccines (mRNA/DNA)	Genetic material encoding antigen is delivered; host cells transiently produce antigen to stimulate immune response.	SARS-CoV-2 (COVID-19 mRNA vaccines)	2020 (SARS-CoV-2)
Bacterial vectored	Live bacteria are genetically engineered to express heterologous antigens and act as delivery systems	Experimental	–
Antigen-presenting cell	Patient-derived dendritic cells are loaded with antigen ex vivo and re-infused to stimulate adaptive immunity (mainly therapeutic use)	Experimental (mainly cancer immunotherapy platforms)	–

## Conclusion

The recent advances in vaccine platforms indicate a transformative period in the field of vaccinology. These novel platforms have the potential to provide personalised cancer vaccines, multivariant vaccines, and enhanced pandemic preparedness and response. Vaccines are among the most effective tools for preventing outbreaks and saving lives. However, equitable access to vaccines and ensuring that they reach underprivileged, yet high-risk populations remain critical. Finally, as vaccinologists, we are accountable for creating a pro-vaccine environment where immunisation is the norm, vaccines are accessible, and delivery is equitable.
